# Why *Naunyn–Schmiedeberg’s Archives of Pharmacology* abandons traditional names of drug classes

**DOI:** 10.1007/s00210-021-02111-4

**Published:** 2021-06-23

**Authors:** Roland Seifert, Bastian Schirmer

**Affiliations:** Institute of Pharmacology, Hannover Medical School, Carl-Neuberg-Str. 1, 30625 Hannover, Germany

*Naunyn–Schmiedeberg’s Archives of Pharmacology* is the oldest existing pharmacological journal, celebrating its 150^th^ anniversary in 2023. In the journal, we place a lot of emphasis on good scientific traditions such as rigorous and fair peer review. We place science itself in the center and not its perceived “priority” or “impact”. We also encourage discussion of weaknesses of studies and do not expect authors to submit “perfect stories”.

However, traditions can also be deleterious if they do not adapt to the progress in scientific knowledge. And we do have such a case now and must adapt: Pharmacological nomenclature, particularly designations of drug classes, have continuously evolved over decades and are deeply rooted in pharmacological textbooks, the scientific literature, and, most importantly, in scientific language. Most names of drug classes are rather descriptive and often focus on clinical indications. Thus, drug class names rarely describe the precise mechanisms of action of drugs which, when the terms were coined, were mostly unknown. Moreover, historically, very little thought was given on consistence of drug class designations among subfields. For example, the prefix “anti_” is used for multiple drug classes acting against a certain disease, without specifying the mechanism of action. In addition, receptor antagonists are inconsistently labeled as “anti_,” “_blockers,” or “_lytics.” Terminology was taken for granted. Three recent articles provide an overview of the problems associated with traditional drug class nomenclature (Seifert 2018; Seifert and Schirmer 2020, 2021).

Over time, an increasing mismatch between terminology and scientific facts evolved. Firstly, in many cases, we now better understand the mechanisms of action of drugs (Seifert 2019, 2021). Secondly, in many cases, drug uses have changed moderately or radically. For example, some “antidepressants” are effective in polyneuropathy, and certain “antidiabetics” are effective in chronic heart failure and chronic kidney disease (Seifert 2019, 2021).

Based on these developments, 3 years ago, one of the authors of this Editorial proposed to critically rethink pharmacological nomenclature (Seifert 2018). Two years later, the authors of this Editorial proposed a simple nomenclature for psychoactive drugs in *Naunyn–Schmiedeberg’s Archives of Pharmacology* (Seifert and Schirmer 2020) and earlier this year, we provided arguments why one of the most deeply rooted terms in pharmacology, i.e., “antibiotics,” should be abandoned (Seifert and Schirmer 2020). The feedback from colleagues on these papers was very positive.

In parallel with the publication of the above-cited papers, a national process to restructure the medical curriculum was initiated in Germany, and the German medical community took the opportunity to abandon traditional drug class designations in the medical curriculum and to replace them with mechanistic or chemical terms (https://nklm.de/zend/menu/index, May 24, 2021). This change in nomenclature is now being taught in all medical schools in Germany and will be the basis for the federal medical exams effective spring 2022 (https://www.impp.de/pruefungen/allgemein/gegenstandskataloge.html, May 24, 2021). A textbook in German language fully adopting the nomenclature change has been recently published (Seifert 2021). An English version of the textbook, still just partially implementing the nomenclature changes, was published in 2019 (Seifert 2019). Because internationally, the textbook was very well received by professors and students for pharmacology curricula, a second edition of the textbook, fully implementing the nomenclature change, will now be prepared. Students enthusiastically embrace the new nomenclature as assessed by student evaluations because it is logical, avoids misunderstandings, and facilitates learning of pharmacology as well as the assignment of drug classes to new indications. Most importantly, from the perspective of students, a rational drug class nomenclature facilitates wording of fair and precise exam questions, specifically multiple-choice questions. From the physician–patient communication aspect, the new nomenclature is also useful because it avoids lengthy explanations, why, e.g., a patient should take an “antidepressant” for treating polyneuropathy.

At an online conference of the Nomenclature Committee of the *International Union of Pharmacologists (IUPHAR)* in April 2021, the nomenclature issue was presented and positively received as well. In addition, among editors of *Naunyn–Schmiedeberg’s Archives of Pharmacology*, the nomenclature proposal received positive comments. It is agreed among authors and editors of *Naunyn–Schmiedeberg’s Archives of Pharmacology* that literature searches with traditional terms such as “antidepressants,” “antibiotics,” “antipsychotics,” and “non-steroidal anti-inflammatory drugs” become increasingly difficult and imprecise (Seifert 2018, Seifert and Schirmer 2020). It is also agreed among all stakeholders of *Naunyn–Schmiedeberg’s Archives of Pharmacology* that changing an imprecise but long-established drug nomenclature in the scientific literature is difficult and cannot be accomplished immediately. Rather, it is a complex and continuous cultural process that takes time. However, doing nothing just because it is laborious is not an option in this case.

As with all cultural processes, the question is, when to start it? Should we wait, until *IUPHAR* has formally agreed on a new nomenclature? This process can take many years. Or should we start now as a kind of pilot project? It is a fact that at the level of teaching, pharmacology textbooks and exam questions, the process has already started, and the next generation of medical doctors and pharmacologists will be familiar with the new nomenclature from their training. But the next generation of medical doctors and pharmacologists will face the traditional nomenclature in the scientific literature and, of course, in many, if not most, internet resources. And established doctors and scientists educated in the old system might have communication problems.

Therefore, we feel that it is now high time to begin implementing the new pharmacological nomenclature not only in pharmacology textbooks but also in pharmacological journals. And this journal will make the first step. It was agreed with the Publisher of *Naunyn–Schmiedeberg’s Archives of Pharmacology*, Springer Nature, that the nomenclature switch must not be abrupt but slow and smooth. This is important for allowing all involved parties, authors, editors, reviewers, and readers alike, a hopefully hick-up-free transition period.

Specifically, we require that authors of all future submissions to *Naunyn–Schmiedeberg’s Archives of Pharmacology* principally use modern drug class nomenclature, either in the unabbreviated from or (preferably) abbreviated form. In general, readability of abbreviated drug class terms is better. In parentheses, authors are encouraged to use the traditional terms to avoid communication problems and ensure searchability of their papers in abstracting and indexing databases in the transition period. Most importantly, authors are not only required to implement the new nomenclature in the manuscript text but also in the abstract. Abstracts are globally and freely available to the scientific community in publication databases and not subject to subscription barriers. Thus, abstracts have great importance for global dissemination of knowledge, key concepts and terminology. It follows that use of modern drug nomenclature use will predominantly be propagated via abstracts. Manuscripts that do not abide to these new editorial guidelines will be administratively rejected and returned to authors for amendment.

We realize that initially, it will require some work from the side of authors to implement the required nomenclature changes, specifically since *Naunyn–Schmiedeberg’s Archives of Pharmacology* is the first and currently only journal addressing this highly important topic. However, we are confident that our authors will soon appreciate that the precision and accuracy of their articles improve and that they can convey their scientific messages in a better and more precise manner.

The pilot project-type implementation of the nomenclature change in this journal is also important for *IUPHAR* and other pharmacological journals. In this way, these players will receive “*real-world*” feedback on which aspects of the nomenclature are good and practical and which aspects need revision.

Below, authors will find a table (Table 1) in which selected traditional drug terms are listed. In the future, for all future submissions to the journal, these terms must be avoided as primary terms in the abstract or manuscript text. Traditional terms should only be used in parentheses after the modern pharmacological terms (unabbreviated or preferably, abbreviated) have been introduced as primary terms. Table 1 also contains a number of problematic terms that should not be used in the future, mainly because they are ambiguous and not clearly defined. Rather, authors should specify the exact drug class they are referring to.

**Table. 1:** List of traditional drug class designations and their proposed mechanistic (or chemical) replacements

Traditional drug class designation	Mechanistic drug class designation (unabbreviated)	Mechanistic drug class designation (abbreviated)
Alpha blocker	α_x_-Adrenergic receptor antagonist	α_x_AR antagonist
Aldosterone antagonist	Mineralocorticoid receptor antagonist	MCR antagonist
Antiandrogen, antiestrogen	Unclear definition, comprises receptor antagonists and enzyme inhibitors; do not use term	Specify exact drug class you are referring to (e.g., AR antagonist, ER antagonist)
Antibiotic	Antibacterial drug	Not applicable
Antiarrhythmic	Multiple mechanisms; also proarrhythmic and local anesthetic effects; do not use term	Specify exact drug class you are referring to (e.g., SCB, HCN4 channel blocker, CCB)
Anticholinergic	Acetylcholine receptor antagonist	AChR antagonist
Antidepressant	Norepinephrine/serotonin enhancer	NE/5-HT enhancer
Antiemetic	Diverse mechanisms and indications; do not use term	Specify exact drug class you are referring to (e.g., CB_1_R agonist; D_2_R antagonist, H_1_R antagonist, 5-HT_3_R antagonist)
Antiepileptic (anticonvulsant)	Diverse mechanisms and indications; do not use term	Specify exact drug class you are referring to (e.g. SCB, CCB)
Antihistamine	Histamine receptor antagonist	H_x_R antagonist
Antipsychotic (neuroleptic)	Antagonist at multiple G-protein-coupled receptors	mGPCR antagonist
Anxiolytic	Unclear meaning; do not use term	Specify exact drug class you are referring to (e.g., mGPCR antagonist, benzodiazepine, SSRI)
Atypical antipsychotic (second-generation antipsychotic)	Unclear definition; do not use term	Specify exact drug you are referring to (e.g., clozapine, olanzapine)
Beta blocker	β_x_-Adrenergic receptor antagonist	β_x_AR antagonist
Betasympathomimetic	β_x_-Adrenergic receptor agonist	β_x_AR agonist
Biological	Suggestive term (“bio” is good for you and has few adverse drug reactions), multiple mechanisms; do not use term	Specify exact drug class you are referring to (e.g., TNF inhibitor, IL-5 inhibitor)
Calcimimetic	Allosteric modulator of the calcium-sensing receptor	Allosteric CaSR modulator
Chemotherapeutic	Ambivalent meaning, do not use term	Specify exact drug class you are referring to (classic cytostatic drug, antibacterial drug, antiviral drug)
Calcium antagonist	Calcium channel blocker	CCB
Corticosteroid (cortison, steroid)	Glucocorticoid receptor agonist	GCR agonist
Depolarizing muscle relaxant	Nicotinic acetylcholine receptor agonist	nAChR agonist
Disease-modifying antirheumatic drug (DMARD)	Not clearly defined, multiple mechanisms; do not use term	Specify exact drug you are referring to (e.g., MTX, ciclosporin, 6-MP, hydroxychloroquine)
Fibrate	Peroxisome proliferator-activated receptor-α agonist	PPAR-α-agonist
Gliflozin	Sodium/glucose transporter 2 inhibitor	SGLT-2 inhibitor
Gliptin	Dipeptidyl peptidase-4 inhibitor	DPP4 inhibitor
Heart glycoside (cardiac glycoside, digitalis glycoside)	Na^+^/K^+^ -ATPase inhibitor	NKA inhibitor
Hypnotic	Unclear definition, do not use term	Specify exact drug class you are referring to (e.g., benzodiazepines, Z substances, barbiturates)
Indirect parasympathomimetics	Acetylcholine esterase inhibitors	AChE inhibitors
Insulin sensitizer	Comprise diverse groups of drugs with beneficial and deleterious effects; do not use term	Specify exact drug class you are referring to (e.g. biguanides, PPAR-γ agonists)
Local anesthetic	Sodium channel blocker	SCB
Loop diuretic	Na^+^/K^+^/2Cl^−^ cotransporter inhibitor	NKCC inhibitor
Mood stabilizer	Group of very different drugs, unclear definition; do not use term	Specify exact drug you are referring to (e.g., lithium lamotrigine)
Non-depolarizing muscle relaxant	Nicotinic acetylcholine receptor antagonist	nAChR antagonist
Non-opioid analgesic	Negative definition of heterogenous group of drugs; do not use term	Specify exact drug class you are referring to (e.g., COX inhibitors, p-aminophenols, pyrazolones)
Non-steroidal anti-inflammatory drug (NSAID) or non-steroidal antirheumatic drug (NSAR)	Cyclooxygenase inhibitor	COX inhibitor
Opioid analgesic	μ-Opioid receptor agonist	MOR agonist
Oral antidiabetic	Not clearly defined, do not use term	Specify exact drug class you are referring to (e.g., sulfonylureas, biguanides, SGLT-2 inhibitors)
Parasympatholytic	Muscarine receptor antagonist	M_x_R antagonist
Parasympathomimetic	Muscarine receptor agonist	M_x_R agonist
RAS (RAAS) inhibitor (blocker)	Comprises drug classes with different mechanisms; do not use term	Specify exact drug class you are referring to (e.g., AT_1_R antagonists, ACE inhibitors, renin inhibitors, MCR antagonists)
Sartan	Angiotensin AT_1_ receptor antagonist	AT_1_R antagonist
Setron	5-Hydroxytryptamine-3 receptor antagonist	5-HT_3_R antagonist
Statin	3-Hydroxy-3-methylglutaryl-coenzyme A-reductase inhibitor	HMG-CoA reductase inhibitor
Sympatholytic and sympathomimetic	Ambiguous meaning; do not use term. Accordingly, the term indirect sympathomimetic should also be avoided	Specific exact drug class you are referring to (e.g., α_2_AR agonists; α_2_AR antagonists)
Thiazide diuretic	Na^+^/Cl^−^ cotransporter inhibitor	NCC inhibitor
Thyreostatic	Thyroid peroxidase inhibitors	TPO inhibitors
Triptan	5-Hydroxytryptamine-1_B/D_ receptor agonist	5-HT_1B/D_R agonist
Typical antipsychotic (first-generation antipsychotic)	Unclear definition; do not use term	Specify exact drug you are referring to (e.g., haloperidol, flupentixol)
Uricosuric	Uric acid transporter 1 inhibitor	URAT1 inhibitor
Uricostatic	Xanthine oxidase inhibitor	XO inhibitor

Traditional drug classes are listed in alphabetical order in the left column. In the center column, unabbreviated replacements are listed, and the right column shows abbreviated replacements. Please note that the list of traditional terms that should be avoided in the future is not yet complete. It focuses on some of the most commonly used terms. Subscript *x* in receptors stands for any given receptor subtype. Drugs acting at receptors are designated as *agonists, antagonists*, *or allosteric modulators*. Dugs inhibiting enzyme are designated as *enzyme inhibitors* and drugs blocking ion channels are designated as *channel blockers*

Authors should note that the list of traditional terms and translation into modern terminology and the list of problematic terms provided in Table 1 is not comprehensive. It just lists some of the most important traditional terms currently used in the literature. Many additional terms can be deduced from this table. Abbreviations for receptors, enzymes, transporters, and ion channels should follow *IUPHAR* rules (https://www.guidetopharmacology.org/nomenclature.jsp, May 24, 2021). For more details, authors are referred to previous publications on terminology of drug classes (Seifert 2018, 2019, 2021; Seifert and Schirmer 2020, 2021). A comprehensive discussion of many more problematic pharmacological terms will be the topic of a future publication to be submitted to this journal.

We encourage our authors and readers to provide feedback on the proposed nomenclature changes to the Editor-in-Chief of *Naunyn–Schmiedeberg’s Archives of Pharmacology*. Any suggestions for improvement of the nomenclature are most welcome. After all, it is a cultural process that must be accepted by the entire scientific and medical community to be effective.

To conclude, your papers will become cutting-edge both scientifically and with respect to modern terminology if you follow our editorial guidelines. And your critical pharmacological thinking will be sharpened as well. Please keep in mind that language shapes thinking and vice versa. And for pharmacology, both aspects should be as precise as possible.

## References

[CR1] Seifert R (2018). Rethinking pharmacological nomenclature. Trends Pharmacol Sci.

[CR2] Seifert R (2019) Basic knowledge of pharmacology, Textbook. Springer, Heidelberg. ISBN 978–3–030–18899–3

[CR3] Seifert R, Schirmer B (2020). A simple mechanistic terminology of psychoactive drugs: a proposal. Naunyn Schmiedebergs Arch Pharmacol.

[CR4] Seifert R (2021) Basiswissen Pharmakologie, Second Edition. Springer, Heidelberg. ISBN 978-3-662-60503-5

[CR5] Seifert R, Schirmer B (2021) A case to stop the use of the term ‘antibiotics’. Trends Microbiol. S0966–842X(21)00093–7. 10.1016/j.tim.2021.03.017 10.1016/j.tim.2021.03.017 33895061

